# Characterizing Active Ingredients of eHealth Interventions Targeting Persons With Poorly Controlled Type 2 Diabetes Mellitus Using the Behavior Change Techniques Taxonomy: Scoping Review

**DOI:** 10.2196/jmir.7135

**Published:** 2017-10-12

**Authors:** Mihiretu M Kebede, Tatjana P Liedtke, Tobias Möllers, Claudia R Pischke

**Affiliations:** ^1^ Prevention and Evaluation Leibniz Institute for Prevention Research and Epidemiology Bremen Germany; ^2^ Institute of Public Health Department of Health Informatics University of Gondar Gondar Ethiopia; ^3^ Faculty of Health Sciences Public Health University of Bremen Bremen Germany; ^4^ Department of Nursing and Health Sciences Fulda University of Applied Sciences Fulda Germany; ^5^ Leibniz Institute for Prevention Research and Epidemiology Prevention and Evaluation Bremen Germany

**Keywords:** type 2 diabetes, telemedicine, mobile health, telehealth, eHealth, mHealth

## Abstract

**Background:**

The behavior change technique taxonomy v1 (BCTTv1; Michie and colleagues, 2013) is a comprehensive tool to characterize active ingredients of interventions and includes 93 labels that are hierarchically clustered into 16 hierarchical clusters.

**Objective:**

The aim of this study was to identify the active ingredients in electronic health (eHealth) interventions targeting patients with poorly controlled type 2 diabetes mellitus (T2DM) and relevant outcomes.

**Methods:**

We conducted a scoping review using the BCTTv1. Randomized controlled trials (RCTs), studies with or pre-post-test designs, and quasi-experimental studies examining efficacy and effectiveness of eHealth interventions for disease management or the promotion of relevant health behaviors were identified by searching PubMed, Web of Science, and PsycINFO. Reviewers independently screened titles and abstracts for eligibility using predetermined eligibility criteria. Data were extracted following a data extraction sheet. The BCTTv1 was used to characterize active ingredients of the interventions reported in the included studies.

**Results:**

Of the 1404 unique records screened, 32 studies fulfilled the inclusion criteria and reported results on the efficacy and or or effectiveness of interventions. Of the included 32 studies, 18 (56%) were Web-based interventions delivered via personal digital assistant (PDA), tablet, computer, and/or mobile phones; 7 (22%) were telehealth interventions delivered via landline; 6 (19%) made use of text messaging (short service message, SMS); and 1 employed videoconferencing (3%). Of the 16 hierarchical clusters of the BCTTv1, 11 were identified in interventions included in this review. Of the 93 individual behavior change techniques (BCTs), 31 were identified as active ingredients of the interventions. The most common BCTs identified were instruction on how to perform behavior, adding objects to the environment, information about health consequences, self-monitoring of the outcomes and/or and prefers to be explicit to avoid ambiguity. Response: Checked and avoided of a certain behavior Author: Please note that the journal discourages the use of parenthesis to denote either and/or and prefers to be explicit to avoid ambiguity. Response: Checked and avoided “and/or” and prefers to be explicit to avoid ambiguity. Response: Checked and avoided, and feedback on outcomes of behavior.

**Conclusions:**

Our results suggest that the majority of BCTs employed in interventions targeting persons with T2DM revolve around the promotion of self-regulatory behavior to manage the disease or to assist patients in performing health behaviors necessary to prevent further complications of the disease. Detailed reporting of the BCTs included in interventions targeting this population may facilitate the replication and further investigation of such interventions.

## Introduction

The global burden of diseases has shifted from communicable diseases to noncommunicable diseases (NCDs) due to interrelated nutritional, sociodemographic, and epidemiological transitions [1]. Deaths attributable to NCDs are expected to rise by 15% between 2010 and 2020 [2,3]. Type 2 diabetes mellitus (T2DM) is one of the major NCDs. Globally, around 415 million people are living with diabetes [4], and the global obesity epidemic [3] has increased its importance for global health. This number is expected to rise to 642 million by 2040. Hence, diabetes has become one of the largest global health emergencies of the 21st century [4].

As a result of advances in information and communication technology (ICT), mobile phones and the Internet are increasingly playing a role in interventions for health promotion and in those aimed at preventing and managing diseases [5]. These technologies may help patients perform behavior necessary for disease management and lifestyle modification and may support long-term treatment. Engaging patients in the care continuum using technological support to improve treatment outcomes and enhancing communication between patients and providers are effective interventions [6,7].

An increasing number of effective ICT applications are currently employed by health providers to improve health behaviors and manage disease outcomes in persons with T2DM [8-13]. Electronic health (eHealth) is the use of ICT for health [14]. Eysenbach defined eHealth as an “intersection of medical informatics, public health, and business, referring to health services and information delivered or enhanced through the Internet and related technologies” [15]. Applications such as telemedicine, videoconferencing, Web-based applications, tailored and untailored text messaging, mobile phone apps, biometric sensors, wearable devices, and Internet-based interactive support systems are currently used for the management of T2DM and to support the adoption of a healthier lifestyle [8,16-21].

Several outcome measures were employed in studies investigating the effectiveness of eHealth interventions targeting persons with T2DM [8]. Blood glucose and hemoglobin A_1c_(HbA_1c_) levels and the incidence of hypoglycemic events are often used as objective primary outcome measures in randomized controlled trials (RCTs) [22-27]. The frequency or rate of T2DM-related complications, adherence to self-care, and prescribed medications are also used to evaluate intervention effectiveness [28]. HbA_1c_ as an outcome measure is relatively well standardized and widely employed in research [8,28,29]. In contrast, measures for assessing changes in lifestyle, quality of life, and other psychosocial outcomes vary substantially [30]. In several studies, the Short Form Health Survey (SF-36) is used to measure quality of life [31,32]. However, other studies prefer using the Problem Areas in Diabetes Scale [20] or the Diabetic Quality of Life (DQoL) [33] questionnaire. Measuring the effectiveness of interventions requires identifying the outcomes of interventions and the tools used to measure the outcomes. Identifying the outcomes facilitates the comparison and syntheses of evidence across multiple interventions. While results of systematic reviews of randomized trials and observational studies suggest that participation in eHealth interventions leads to improvements in disease-related outcomes and health behaviors as well as a reduced risk for complications, the active ingredients of these interventions remain unclear [34,35]. The lack of homogeneity of measurements and the complexity of identifying and summarizing active ingredients of interventions make synthesizing and replicating the evidence a challenging task [35,36]. This is further complicated by poor descriptions of intervention content often available in scientific publications [37]. Therefore, adding to existing research findings, synthesis of evidence, and reliable implementation of interventions is limited [35,36].

Several models and taxonomies have been developed to help describe intervention content and simplify reporting of the effects of behavioral interventions. For example, using the Behavioral Change Wheel (BCW), researchers can organize content and components of behavioral interventions into 9 intervention functions: education, persuasion, incentivization, coercion, training, enablement, modeling, environmental restructuring, and restrictions [38]. The BCW model provides a systematic way of classifying behavioral change interventions using the 9 intervention functions and 7 policy categories. To translate the general intervention functions into specific techniques that were employed in a given intervention to change behavior, Michie et al [39] recommend the application of the Behavior Change Techniques Taxonomy Volume 1 (BCTTv1) (www.behaviourchangewheel.com/about-wheel). The Effective Practice and Organization of Care (EPOC) taxonomy [40] was used in a systematic review by Tricco and colleagues [41] to categorize and aggregate the effectiveness of 142 quality improvement studies in diabetes. Categories included education of patients, promotion of self-management, and reminder systems [41]. Both the EPOC taxonomy and the BCW model of intervention content evaluation are considered important hallmarks of a more reliable content analysis and the development and use of a common language for describing intervention components. However, a recent systematic review including 23 randomly sampled studies of 142 interventions demonstrated significant limitations of the EPOC taxonomy. Specifically, the level of detail with regard to content and the mode of delivery of interventions were not well represented when using the taxonomy [42]. Similarly, Drake and colleagues [43] called for a standardization of intervention content analysis. They pointed out difficulties they encountered when synthesizing the literature due to a lack of common language and a reliable model for analyzing intervention content [43]. Reliable content analysis of interventions and synthesis of evidence have been challenging due to poorly described behavioral interventions, a general inconsistency of terminologies across interventions, and the lack of replicable intervention content analysis methodology [36,44,45]. We believe that in comparison to the BCW model and the EPOC taxonomy, the BCTTv1 appears to be a more comprehensive, detailed, reliable, and useful tool in assisting researchers in retrospectively identifying the active ingredients of interventions, particularly behavioral interventions. The BCTTv1 includes 93 behavior change techniques considered to be effective for behavior change and 16 hierarchical clusters [44].

The BCTTv1 has been validated and is used to design and retrospectively evaluate and aggregate effect sizes of eHealth and other behavioral health interventions [46]. This is of particular importance because some evidence suggests that when theory in delineating intervention outcomes is used as a foundation for intervention design, the impact of interventions on those outcomes increases. Results of several studies suggest that eHealth interventions targeting persons with T2DM that are grounded in theory are associated with positive clinical, psychological, and behavioral outcomes such as reductions in HbA_1c_ levels, systolic blood pressure, cholesterol levels, and depression and increases in physical activity [47-51]. To our knowledge, the BCTTv1 has never been applied to evaluate eHealth interventions targeting persons with poorly controlled T2DM. Hence, this scoping review was initiated to identify relevant outcome measures reported in studies examining the effects of eHealth interventions in persons with poorly controlled T2DM and characterize the contents of the interventions targeting this particular population using the BCTTv1.

## Methods

### Framework

Throughout this paper, we follow the definition of eHealth by Eysenbach [15]. We use eHealth interventions in T2DM as a term to refer to all mobile Health (mHealth) interventions—those delivered via personal digital assistant (PDA), tablet, computer, Internet, and other forms of ICT—implemented to improve the management and outcomes of T2DM.

To address the objectives of this scoping review, we followed the 5 steps described in the framework by Arksey and O’Malley [52]: (1) identifying the research question, (2) identifying relevant studies, (3) selecting relevant studies, (4) charting data from the selected studies, and (5) summarizing and reporting the results [52]. Unlike systematic reviews, scoping reviews do not quantitatively aggregate the evidence but rather collate and summarize the evidence by mapping the related literature and examining the extent, breadth, nature, and characteristics of the available evidence [52]. Levac et al [53] recommended additional substeps to deal with the challenges encountered while conducting scoping studies. The details of the main challenges in each stage and the recommended substeps can be read elsewhere [53].

### Stage 1: Identifying the Research Question

The research questions were developed after a rapid scan of the eHealth literature regarding the areas of prevention, self-management, and long-term medical care for persons with T2DM. We hypothesized that eHealth interventions play an important role in supporting patients who are under diabetic care. We also hypothesized that eHealth interventions targeting persons with T2DM include behavioral components. To search the relevant evidence for our hypothesis, we formulated the following research questions: Which outcome measures are used to assess the effectiveness of eHealth interventions in poorly controlled T2DM patients? What are the active ingredients of the eHealth interventions in poorly controlled T2DM?

### Stage 2: Identifying Relevant Studies

**Figure 1:**
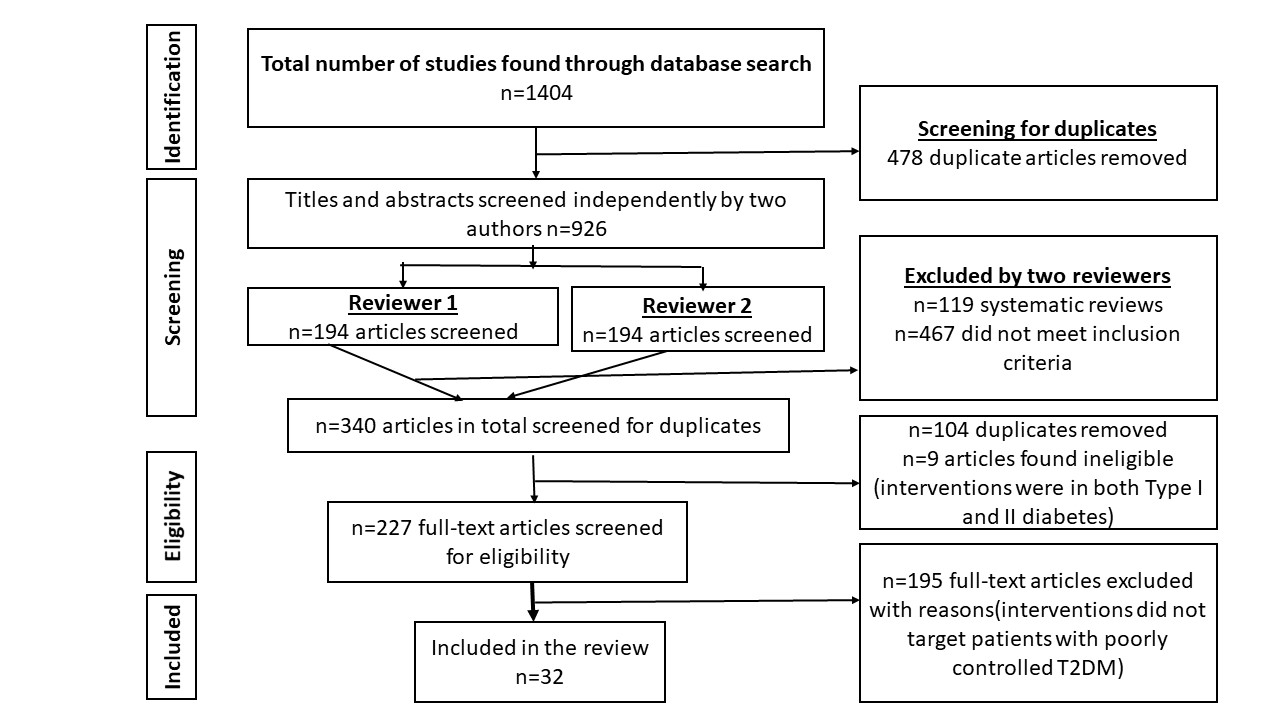
Preferred Reporting Items for Systematic Reviews and Meta-analyses flowchart for database search and study selection.

PubMed, PsycINFO, and Web of Science were searched for relevant studies. During a preliminary search, we did not observe major differences in search results when using Excerpta Medica (EMBASE), Cumulative Index to Nursing and Allied Health Literature (CINAHL), and Cochrane Library. Therefore, we concluded that PubMed and Web of Science covered the relevant articles. Articles containing results pertaining to eHealth interventions targeting patients with poorly controlled T2DM (HbA_1c_ ≥7.0%) published in peer-review journals from January 1990 to June 2016 were considered as potentially relevant for the review. To be included in the review, articles had to report findings of studies with quasi-experimental or pre-/post-designs or of RCTs and had to have a focus on eHealth interventions and poorly controlled T2DM. Articles were excluded if they were published in languages other than English, if only titles were available, and if they were study protocols for future or ongoing evaluations of eHealth interventions. The screening process and identification of the relevant studies are shown in Figure 1.

The key word search strategy employed to identify relevant literature is described in Multimedia Appendix 1. All search results of PubMed, PsycINFO, and Web of Science were exported to EndNote version X 7.3 reference software (Clarivate Analytics).

### Stage 3: Study Selection

Two authors (MK and TM) independently examined the titles and abstracts of eHealth intervention studies targeting persons with T2DM to assess their relevance for the review. We used the American Diabetes Association definition of poorly controlled T2DM as having an HbA_1c_ level of ≥7.0% [54,55]. In line with the framework by Arskey and O’Malley [52], a quality appraisal or quality assessment was not performed because it is not essential for scoping studies. Hence, methodological rigor of the published articles was not a criterion for inclusion or exclusion. The titles and abstracts previously selected by the 2 independent reviewers were merged and further screened for duplicates and following predefined inclusion criteria.

### Stage 4: Charting of the Data

#### Preparation of the Data Extraction Form

Levac and colleagues [53] recommend cooperatively developing the data extraction form, an iterative data extraction process, independent extraction of data by multiple authors, and qualitative content analysis. Following this recommendation, a data extraction form was first prepared by MK and CP. The data extraction process and assurance of the quality of data was iterative with frequent updates of the extraction form and the data collected from the studies.

The data extraction spreadsheet (Multimedia Appendices 2 and 3) included the following items:

1. Authors, title, journal, year of publication, issue, volume, study location (identified by the corresponding author’s address and/or the context of the study explained in the methodology)
2. Type of intervention, tailoring or individualization of the intervention, comparator (if any), duration of intervention, theories or models used for designing the intervention
3. Study population, size of the population
4. Aim of the study
5. Study design
6. Outcome measures, measurement tools
7. Results
8. Intervention active ingredients coded using the BCTTv1

#### Independent Data Collection by Reviewers

Three reviewers, MK, TM, and TL, independently collected the data using the extraction form. In addition, CP collected data from 5 randomly selected studies to check the quality of the data previously extracted by MK, TM, and TL. The reliability and quality of the extracted data was also ensured through subsequent meetings, cross-checking of the collected data, discussions to resolve disagreement in data extraction, rereading of the full texts of the papers, refining the extraction form, and revising the collected data.

#### Collaborative Exploration of the Interventions and Outcome Measures and Identification of the Active Intervention Ingredients

This was the main step for answering the research questions and required all reviewers to reach consensus regarding the classification of the type of intervention delivery and content and identification of the outcome measures. Here, the descriptive analytical narrative method was employed [53]. In addition, using thematic content analysis, type of intervention and outcome measures were exclusively categorized by content, nature of outcomes, and context/setting of implementation.

The descriptions of all interventions were analyzed, and active ingredients of the interventions were identified following the BCTTv1 by Michie and colleagues [44].

Emphasis was put on reaching consensus with regard to the labeling of the intervention components according to the taxonomy (Multimedia Appendix 3). MK and TL independently analyzed the contents of the interventions using the taxonomy. Analysis was followed by discussions between MK and TL regarding the coding. When there was disagreement, CP was consulted to reach consensus. Whenever we were indecisive in coding, we used the BCTTv1 coding rules supplement by Presseau and colleagues [42]. When the BCTTv1 and the coding supplement were not clear enough to characterize intervention content, the following 5 coding assumptions were used:

1. If an intervention included an educational component but sufficient detail on the themes and sequence of educational activities was not provided, the intervention was given the labels “information about health consequences” and “instruction on how to perform behavior.”
2. If an intervention included training without providing detail regarding the training, it was labeled as “instruction on how to perform behavior.”
3. If patients in a given intervention received blood glucose or blood pressure measurement devices, Internet services, software applications, computers, mobile phones, and/or airtime services, booklets, or leaflets, the intervention was labeled as “adding objects to the environment.”
4. If an intervention included warning or cautionary messages to raise patients’ consciousness regarding dangers of an unhealthy diet or sedentary behavior or clinical parameters reaching certain values (eg, elevated blood glucose, blood pressure), this was labeled as “salience of consequences.”
5. If motivational messages or calls or motivational interviewing were included in an intervention, the intervention was coded as “social support (emotional).”

### Stage 5: Collating, Summarizing, and Reporting the Results

After charting the relevant data from the studies in spreadsheets, the results were collated and described using summary statistics, charts, figures, and tables. First, the types of eHealth interventions were charted into categories. Second, the outcome measures using studies examining the role of eHealth interventions in poorly controlled T2DM were categorized. Third, by exploring the contents of the intervention and cross-checking them with the definitions and examples of the 93 techniques in the BCTTv1, the active ingredients of the interventions were coded (Multimedia Appendix 3).

## Results

### Study Selection and Characteristics

Keyword searches in PubMed, Web of Science, and PsycINFO resulted in 624, 775, and 5 articles respectively (Multimedia Appendix 1), with a total of 1404 articles.

Removing the duplicates, subsequent screening, and eligibility assessment of the titles and abstracts led to 227 potentially relevant articles. Screening the full texts of these 227 articles and applying the eligibility criteria resulted in 32 studies [6,20,25,31-33,56-81] being included in the review (Figure 1).

**Figure 2:**
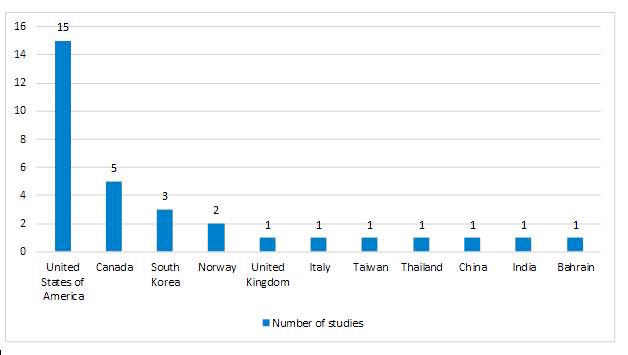
Geographical distribution electronic health intervention studies in poorly controlled type 2 diabetes mellitus.

Geographically, most of the studies included in the review were conducted in the United States (46.9%), followed by Canada (15.6%) and Europe (12.5%) (Figure 2).

Regarding the study design, 16 studies were RCTs, 4 were parallel and 3 cluster RCTs, 3 had pretest/posttest designs, 2 were 3-arm randomized trials, 1 was a prospective randomized trial, and 1 was a nonrandomized controlled intervention.

**Figure 3:**
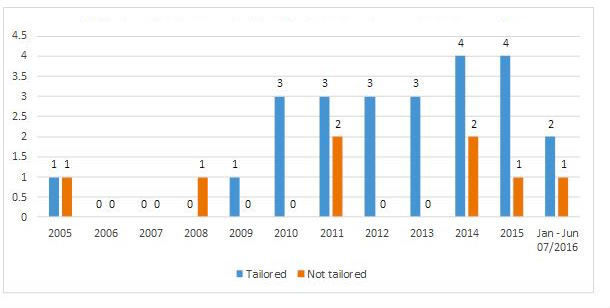
Distribution of tailoring in electronic health interventions.

Among the 32 eHealth interventions investigated in the included studies, 24 (75%) were tailored to the health and behavioral characteristics of the individual patient. According to the evidence (seen in Figure 3), an increasing trend for individualization of intervention content was observed.

Only 8 interventions were designed following theories or models of behavioral change. The theories/models used for designing the interventions were cognitive behavioral therapy [31,78], the reach out problem-solving model [31,78], motivational interviewing [31,61,78], the universal model of behavioral change [6], Green and Kreuter’s PRECEDE-PROCEED model [63], the health belief model [20], the community model [32], and Wagner’s chronic care model [71].

### Modes of Delivery of eHealth Intervention

Of the 32 interventions, 18 (56%) were PDA-, tablet-, computer-, or mobile phone–delivered or Web-based interventions [6,25,31,33,58,60,61,68-71,74-80], 7 interventions (22%) were telehealth interventions delivered via landline telephones [57,59,63,66,67,73,81], 6 (19%) used text messaging [20,56,62,64,65,72], and 1 employed videoconferencing [32].

### Outcome Measures of eHealth Interventions in Poorly Controlled Type 2 Diabetes Mellitus

**Figure 4:**
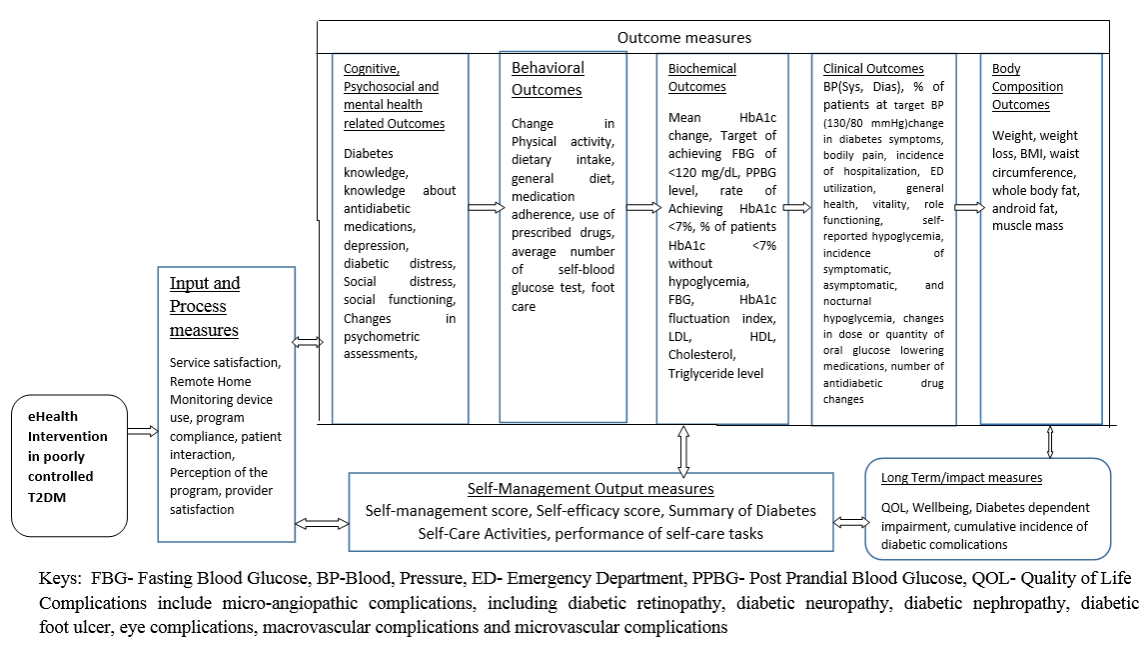
Outcome measures of electronic health effectiveness.

Changes in HbA_1c_ level were used as the primary outcome in the majority (28/32, 88%) of the studies. In addition, outcomes such as changes in lipid profiles (ie, total cholesterol, high-density lipoprotein [HDL], low-density lipoprotein [LDL], and triglyceride levels), changes in dose and quantity of antidiabetic drugs, use of drugs, adherence to treatment, and changes in diabetic knowledge were used as primary outcomes. Examples of secondary outcomes used in the interventions include patient satisfaction, medication adherence, performance of self-care tasks, and quality of life. The detailed list of primary and secondary outcomes employed in the included intervention studies are outlined in Multimedia Appendix 2. The outcome measures were broadly categorized and a framework was then developed (Figure 4) including all outcomes and suggesting pathways between different outcomes.

### Acceptance and Use of Interventions

Outcomes included in this category were service satisfaction, remote home-monitoring device use, program compliance, patient interaction, perception of the program, and provider satisfaction.

### Self-Management

This category included behaviors pertaining to disease self-management (ie, self-management score, summary of diabetes self-care activities, and performance of self-care tasks).

### Outcome Measures

The outcome measures were the intermediate outputs that were considered to lead to the long-term effects of the interventions.

#### Cognitive and Psychosocial Outcomes

In the reviewed literature, diabetes knowledge, self-efficacy score, and knowledge about antidiabetic medications were used to assess the cognitive outcomes of eHealth interventions among poorly controlled T2DM patients. Outcomes such as depression, diabetic distress, social distress, social functioning, and changes in psychometric assessments were used to assess the effects of eHealth interventions on psychosocial outcomes.

#### Behavioral Outcomes Regarding Health and Self-Management Behavior

Outcomes including in this category were any changes in physical activity, dietary intake, general diet, medication adherence, use of prescribed drugs, average number of self-blood glucose tests, and self-reported foot care reported in the literature.

#### Glycemic Control Markers

Changes in glucose levels were measured by the mean HbA_1c_ change, achieving a fasting blood glucose of <120 mg/dL, postprandial blood glucose level of <180 mg/dL, HbA_1c_ <7%, fasting blood glucose levels of 80 to 130 mg/dL, HbA_1c_ fluctuation index, and percentage of patients with an HbA_1c_ <7% without hypoglycemia.

#### Biological Markers and Other Clinical Outcomes

Outcomes used to measure the effectiveness of eHealth interventions in poorly controlled T2DM patients included in this category were the following: blood pressure (systolic and diastolic); percentage of patients at the target blood pressure (130/80 mm Hg); change in diabetes symptoms; LDL, HDL, cholesterol, and triglyceride levels; change in incidence of hospitalization; emergency department utilization; self-reported hypoglycemia; incidence of symptomatic, asymptomatic, and nocturnal hypoglycemia; changes in dose or quantity of oral glucose lowering medications; and number of antidiabetic drug changes.

#### Body Composition Outcomes

Weight, weight loss, body mass index, waist circumference, whole body fat, android fat, and muscle mass were the main outcome measures reported in the literature and included in this category.

### Long-Term Outcomes

Long-term effects of the intervention were quantified by changes in diabetic quality of life, bodily pain, general health, vitality, role functioning, general well-being, diabetes dependent impairment, and the cumulative incidence of diabetic complications, including incidence of microangiopathic complications (ie, diabetic retinopathy, diabetic neuropathy, diabetic nephropathy, diabetic foot ulcer, eye complications, macrovascular complications, microvascular complications).

In most of the studies (25 out of 32), a statistically significant change in HbA_1c_ percentage was used as a primary measure of eHealth intervention effectiveness in changing glucose levels in persons with poorly controlled T2DM. However, changes in diabetes knowledge [69], knowledge about antihyperglycemic medications, patient-reported medication decisional conflict [61], and cumulative incidence of diabetic complications [82] were also used as a primary outcome measures for assessing eHealth intervention effectiveness. In addition, achieving the target of fasting blood glucose <120 mg/dL, fasting and postprandial blood glucose levels [64], changes in physical functioning and role limitations [32], self-efficacy, medication adherence [20], proportion of patients achieving HbA_1c_ <7% without hypoglycemia [65], adherence to treatment prescriptions, and use of drugs [72] were used to determine intervention effects.

These outcomes were combined in the framework displayed in Figure 4. This framework was developed after careful examination of the nature of each outcome and hypothesizing its relationship in the pathway.

### Characterizing the Contents of Interventions Using the Behavior Change Techniques Taxonomy Volume 1

**Figure 5:**
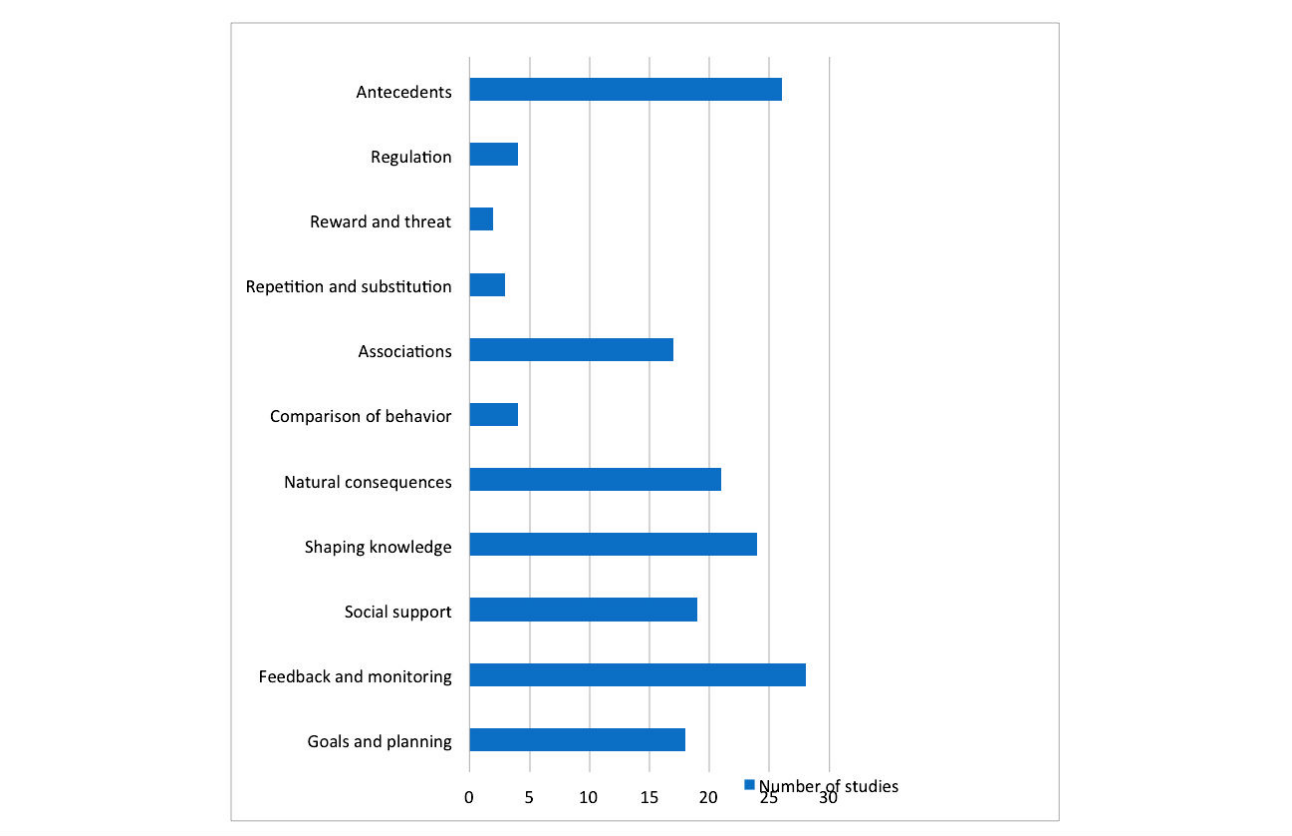
Frequency distribution of Behavior Change Techniques Taxonomy Volume 1 hierarchical clusters coded for 32 interventions.

The types of behavior change techniques (BCTs) identified in the selected interventions are described in Multimedia Appendix 4. All of the 32 interventions included multiple BCTs. Of the 16 overarching thematic categories, 11 (69%) were addressed in interventions: goals and planning, feedback and monitoring, social support, shaping knowledge, natural consequences, comparison of behavior, associations, repetition and substitution, reward and threat, regulation, and antecedents (Figure 5). No BCTs from the following 5 overarching categories were identified: comparison of outcomes, identity, scheduled consequences, self-belief, and covert learning. Of the 16 hierarchical clusters of the BCTTv1, feedback and monitoring was included in 27 of the 32 studies.

**Table 1:** Behavior change techniques and number of interventions that included specific behavior change techniques, Behavior Change Techniques Taxonomy Volume 1 hierarchical clusters, and intervention content examples.

BCT^a^	BCTTv1^b^ hierarchical clusters	Examples extracted from descriptions of the interventions	Frequency	Studies that included the BCT
Instruction on how to perform a behavior (4.1)	Shaping knowledge	“Each participant received overall orientation on diabetes management (including how to inject insulin) as well as nutritional and exercise education” [64].	24	[6,20,25,31,32,56,58-60,64-66,68-75,77-80]
Adding objects to the environment (12.5)	Antecedents	“Participants were provided with wireless remote monitoring tools and enhanced patient portal functions to support self- management of diabetes” [6].	23	[6,20,25,31,32,56,57,60,61,64,65,67-70,73-79,81]
Information about health consequences (5.1)	Natural consequences	“If the sum of all high glycemic index foods in the previous 24-hour period is 5 or more servings, then it provided a more educational message regarding high and low glycemic index foods” [63].	21	[6,32,33,56-58,61,63-73,75,78]
Self-monitoring of outcomes of behavior (2.4)	Feedback and monitoring	“...Patients could view their trends over time and make associations between their behaviors and test results” [56].	19	[6,25,31,56,57,60,64,65,67-70,73-79]
Feedback on outcomes of behavior (2.7)	Feedback and monitoring	“If remote home-monitoring alerts are judged by the nurse to be significant, trigger an outbound call to the patient to arrange for a provider visit, additional services, or use of the emergency services, as needed” [81].	17	[6,25,31,56-58,65,67-70,73-76,79,81]
Prompts/cues (7.1)	Associations	“An alarm activates if the blood glucose level falls below 4 mmol/L” [74].	16	[6,33,56,62-64,67,68,71-76,81]
Goal setting (outcome) (1.3)	Goal and planning	“With health coaching assistance, clients determined health-related goals...” [80].	9	[6,20,25,31,59,61,64,66,71,80]
Social support (unspecified) (3.1)	Social support	“Internet browser was set to a diabetes education website designed for the study, containing links to several websites with vetted content related to diabetes self-management including sites that facilitated peer-sharing and mutual support” [68].	9	[33,56,57,60,62,67-69,80]
Action planning (1.4)	Goal and planning	“Patients received an electronic action plan every 2.5 months to support improved diabetes self-management and to serve as previsit summaries for physician office visits” [69].	8	[6,25,61,62,69-71,80]
Self-monitoring of behavior (2.3)	Feedback and monitoring	“The intervention contained email, text, and website with self-regulation, self-monitoring, and assessment functions...food/nutrition, exercise, emotion, and general health care were included” [33].	8	[32,33,60,69-71,78,80]
Social support (practical) (3.2)	Social support	“The nurse at the health office educated the patient face-to-face according to the physician’s recommendations” [58].	8	[25,31,58,63,70,77,78,80]
Feedback on behavior (2.2)	Feedback and monitoring	“Care manager–participant contacts were used to review progress, reinforce nutritional and lifestyle modifications, and make medication changes” [68].	7	[31,62,63,68,69,74,77]
Social support (emotional) (3.3)	Social support	“Patients received phone calls from diabetic educators on days 3, 7, 14, and 60 after registration for specific barrier education, data explanation, and confidence establishment” [57].	7	[6,31,57,69,71,73,81]
Biofeedback (2.6)	Feedback and monitoring	“...Patients were provided with special blood glucose testing before and after each exercise session” [80].	6	[56,59,64,69,71,80]
Goal setting (behavior) (1.1)	Goal and planning	“The message protocol included encouragement toward self-entered weight loss and exercise goals” [56].	5	[33,56,61,78]
Problem solving (1.2)	Goal and planning	“Text messages were sent to solve problems, support patients’ needs, and improve skill...” [62].	5	[20,57,58,61,62,71]
Salience of consequences (5.2)	Natural consequences	“Education using animations of how diabetes affects how glucose is processed in the body and how different medication classes, foods, and physical activity affect blood sugar. When patients consume high glycemic index foods, they received a slightly more strongly worded message that also gave information about end-organ damage when diabetes remains uncontrolled.” [61].	4	[20,61,63,73]
Demonstration of the behavior (6.1)	Comparison of behavior	“The exercise regimen consisted of a combination of aerobic and resistance exercises of 10-minute duration each, with 5-minute warm-up and cool-down periods... The subjects were encouraged to do this at home daily or on most days of the week” [32].	3	[32,61,64,79]
Discrepancy between current behavior and goal (1.6)	Goal and planning	“Interactive visual displays of facilitated tracking progress toward goals and correlated glucose control with medication compliance or lifestyle changes” [6].	2	[6,80]
Information about antecedents (4.2)	Shaping knowledge	“Patients were required to test glucose whenever they had symptoms related to hypoglycemia and to record their blood glucose readings” [59].	2	[59,77]
Social reward (10.4)	Reward and threat	“If the portion of high glycemic index foods is 0-1, they received a message of congratulations and encouragement to continue the same” [63].	2	[63,76]
Pharmacological support (11.1)	Regulation	“The diabetes status report displays diabetes-related medications—emphasizing the medications most important to risk reduction of diabetes complications” [73].	2	[58,73]
Reduce negative emotions (11.2)	Regulation	“Education on stress management and keeping well and healthy, participants were introduced to their self-care model and gained more confidence in the way they faced life stressors” [66].	2	[66,80]
Restructuring the social environment (12.1)	Antecedent	“...The intervention was designed to improve skills and action plans while contacting the team in anywhere and anytime manner” [62].	2	[62,80]
Review behavior goals (1.5)	Goal and planning	“...Participants set goals and develop specific action plans to address identified barriers or other concerns and identify specific questions and concerns to discuss with their doctor about their medications or making lifestyle changes”[61]	1	[61]
Reduce prompts/cues (7.3)	Associations	“Patients stopped self-monitoring when target blood glucose levels were achieved and resumed self-monitoring prior to quarterly visits and if 3-monthly HbA_1c_ was >53 mmol/L (7.0%)” [25].	1	[25]
Behavioral practice/rehearsal (8.1)	Repetition and substitution	“The technique of progressive muscular relaxation was also taught in one of the sessions, with the advice of practicing this at home whenever the subjects encounter stress” [32].	1	[32]
Behavioral substitution (8.2)	Repetition and substitution	“...Provided education on common foods in their diet which have a high glycemic index, with low/moderate glycemic index food substitutes...” [63].	1	[63]
Habit formation (8.3)	Repetition and substitution	“For the duration of the project, a helper is available at all times in the community centers during the group sessions...” [32].	1	[32]
Graded tasks (8.7)	Repetition and substitution	“The insulin self-titration was based on an individualized stepwise treatment plan which contains a number of discrete successive medication doses (steps)...” [25].	1	[25]
Body changes (12.6)	Antecedent	“Progressive muscular relaxation was taught...” [32].	1	[32]

^a^BCT: behavior change technique.

^b^BCTTv1: Behavior Change Techniques Taxonomy Volume 1.

Of the individual 93 BCTs of the BCTTv1, 31 (33%) were employed in interventions to change behavior to manage poorly controlled T2DM. On average, 6.7 BCTs (SD 2.0) were included in interventions. The BCTs and the specific content of interventions with examples are displayed in Table 1. The maximum number of BCTs included in 1 intervention was 11 and the minimum was 3 (Multimedia Appendix 4).

## Discussion

### Principal Findings

The purpose of this review was to identify the relevant outcome measures reported in studies examining the effects of eHealth interventions in persons with poorly controlled T2DM and characterize the active ingredients of eHealth interventions among persons with poorly controlled T2DM using the BCTTv1.

Most of the studies (25 out of 32) measured the effectiveness of eHealth interventions using a statistically significant change in HbA_1c_ percentage as a primary outcome measure. This is similar to the review reported by Vorderstrasse and colleagues [29]. A review from the Cochrane Library by Pal and colleagues [27] found that all 16 RCTs included in its review used HbA_1c_ percentage as a primary outcome measure of effectiveness. Lipska and Krumholz [83] challenged this glucocentric approach, reporting that the effectiveness indicator of interventions in T2DM is moving away from the historic surrogate marker (ie, HbA_1c_) to cardiovascular outcomes.

The identification of the active ingredients of the behavioral interventions is a basis for synthesizing evidence, building on evidence, and replicating interventions targeting behavioral change. The development and use of the EPOC taxonomy and BCW models contribute to the homogeneity in characterizing the contents of different interventions and in quantifying intervention effects (eg, by aggregating effect sizes). However, we observed that these 2 frameworks were not sufficiently comprehensive to characterize the content of interventions in detail. BCTTv1, in contrast, appeared suitable for in-depth analysis of the active ingredients of interventions. It offered a means of handling heterogeneity and provided a baseline for meta-analysis or the estimation of effect sizes for quantifying effects of behavior change interventions.

In our scoping review, only 31 (33%) of the 93 BCTs were identified in interventions. Similarly, Presseau et al [42] identified less than a quarter of the 93 BCTs in 23 interventions. BCTs such as credible source, reward (outcome), focus on past success remain underused in interventions. Innovative eHealth interventions employing these BCTs need to be tested with regard to their impact in changing patient behavior and affecting T2DM outcomes. Of the 31 BCTs identified in interventions included in this review, the most frequently used were instruction on how to perform behavior, adding objects to the environment, social support (practical), feedback on outcomes of behavior, self-monitoring on outcomes of behavior, and prompts/cues. Van Vugt and colleagues [49] identified BCTs such as providing feedback on performance of behavior, providing information on consequence of behavior, problem solving, and prompts/cues as the most commonly used BCTs in Web-based self-management programs for patients with T2DM. Pal [27] demonstrated that among the most frequently used BCTs, prompt self-monitoring of behavioral outcome and provide feedback on performance were reported to have significant effects on HbA_1c_ levels. However, frequency of inclusion of an individual BCT is neither proof for it significantly improving patient outcomes nor proof of a proper design of interventions [84].

Our study results suggest that, on average, 6.7 BCTs were included per each intervention. The evidence on whether including many BCTs in an intervention improves patient outcomes is not strong. Systematic reviews by Avery and colleagues [50] and Cradock [85] revealed that only 50% and 60%, respectively, of the most frequently used BCTs were associated with reductions in HbA_1c_. An evaluation of diabetes-related apps by Hoppe and colleagues [86] indicated that diabetes mobile phone apps having more BCTs also had significantly higher functionalities and higher user ratings. However, which combination of BCT ingredients had a stronger effect and which BCTs were key moderators of effectiveness in poorly controlled T2DM needs to be further investigated. Customizing eHealth interventions to individual behavioral characteristics and disease progress increases the effectiveness of the intervention [87]. Tailoring or individualizing the communication between patients and providers has gained substantial attention in the past decade. In this review, we observed that more than 75% of the interventions were customized to the individual patient characteristics or needs. In addition, a generally increasing trend of tailored eHealth interventions was noted in the reviewed studies. Strategies used for tailoring vary across studies. Kim [64] and Wayne [80] used pragmatic approaches of tailoring and contextualized the intervention with respect to the individual patient. McFarland and colleagues [67] tailored the intervention to individualize the communication between patients and providers. First, patients self-monitored blood glucose levels by using monitors and transmitted their data using a messaging device. A registered nurse then downloaded the message and contacted the patient via telephone to evaluate whether there were any specific health concerns. Based on specific concerns (eg, with regard to adherence to certain medications or a dietary regimen or hypoglycemic events), patients were given recommendations regarding insulin dosage or lifestyle changes. Ralston and colleagues [71] tailored the Web-based intervention according to the clinical condition of each patient. Accordingly, the care manager responded to specific messages from each patient and reviewed the submitted blood glucose levels of each patient to adjust hypoglycemic medications as needed. Several studies suggest that tailoring may be an effective means of behavioral change and improving self-management skills [88-91]. Tailoring also helps initiate, enhance, and safeguard the partnership between the provider and the patient, increasing shared decision-making and person-centered care which ultimately facilitates the uptake of the desired behavior, such as healthy eating and improved physical activity [92]. However, a recent systematic review reported that there is lack of evidence to suggest tailored eHealth interventions are more effective than nontailored interventions [93]. Therefore, this issue obviously requires more research.

Despite a broad consensus that the use of theories or models to guide the development of interventions leads to greater impact of interventions, the current review showed that only 8 (25%) of the 32 eHealth interventions were theory-based. The finding of our review is consistent with the claim that undertheorization of eHealth interventions and underutilization or an inadequate application of behavioral science and health education theories is still a major issue in the eHealth intervention literature [38,47,84,85,94]. The evidence on effectiveness of designing and implementing interventions through the use of theories is mixed. Some evidence suggests that theory-grounded eHealth interventions are more likely to be associated with positive outcomes of patients with T2DM. Theories can enhance the uptake of the desired behavior by supporting providers and patients to collaboratively set targets, enhance the motivation of intervention participants, and provide a roadmap for behavior and treatment modification [47-51]. The impact and processes by which eHealth interventions influence outcomes are not directly comparable to the impact of pharmacological drugs that are administered into the body and bring a change within a certain period of half-life of the ingredient. eHealth interventions, in part, impact cognitive processes (eg, by improving knowledge) and may help intervention participants internalize the advantages of performing the target behavior, such as improving self-management, dietary, or physical activity behavior, leading to long-term maintenance of these behaviors. Behavior maintenance can then be subsequently linked with changes in biological markers and long-term changes in quality of life and a lower incidence in complications.

### Limitations

Our scoping review had several limitations. The definition of poorly controlled diabetes was based on that of the American Diabetes Association. However, other guidelines, such as the one from the National Institute for Health and Care Excellence, consider an HbA_1c_ level up to 7.5% as a good indicator of glycemic control. This should be taken into consideration while interpreting our results. In some cases, it was challenging to crossmatch intervention contents described in the articles with the BCTs. For example, there were interventions that included motivational messages or calls to induce the uptake of a target behavior. However, motivation was not explicitly described in BCTTv1. In addition, there were interventions with poor descriptions. For instance, interventions provided education but there was no information available regarding the type of education. Coding the poorly described interventions was therefore challenging. Hence, we were forced to develop assumptions to deal with poorly described interventions. Another limitation of our scoping review was that the correlation between the 2 reviewers coding the BCTs was not systematically assessed. Rather, 2 coders independently analyzed contents of interventions, and where they disagreed, a third person was consulted to reach consensus.

### Conclusion

For most interventions, changes in HbA_1c_ levels were reported as a primary measure of effectiveness. Overall, the BCTTv1 appeared practical and helped identify the active ingredients of interventions. Our results suggest that one-third of the 93 BCTs were employed in eHealth interventions targeting persons with poorly controlled T2DM.

Developing theory-based interventions and considering BCTs during the intervention design phase is desirable for obtaining effective interventions and transparently reporting the results of these interventions in the future and possibly in other chronic disease contexts.

## Appendix

Multimedia Appendix 1 Search keywords and output.

Multimedia Appendix 2 Behavioral change technique data extraction form and data.

Multimedia Appendix 3 Extraction form and data.

Multimedia Appendix 4 Behavior change techniques included in electronic health interventions targeting individual behavior change.

## Abbreviations

BCTTv1: Behavior Change Techniques Taxonomy Volume 1
BCW: Behavioral Change Wheel
CINAHL: Cumulative Index to Nursing and Allied Health Literature
DQoL: Diabetes Quality of Life Questionnaire
EMBASE: Excerpta Medica database
EPOC: Effective Practice and Organization of Care
HbA1c: hemoglobin A1c
HDL: high-density lipoprotein
ICT: information and communication technology
LDL: low-density lipoprotein
NCD: noncommunicable disease
PDA: personal digital assistant
RCT: randomized controlled trial
SF-36: Short Form Health Survey
T2DM: type 2 diabetes mellitus
