# Supplementary material for: Characterizing Active Ingredients of eHealth Interventions Targeting Persons With Poorly Controlled Type 2 Diabetes Mellitus Using the Behavior Change Techniques Taxonomy: Scoping Review
Source: J Med Internet Res. 2017 Oct 12;19(10):e348. doi: 10.2196/jmir.7135 (PMC5658649; doi:10.2196/jmir.7135)
Supplement: Multimedia Appendix 1 [file jmir_v19i10e348_app1.pdf]

Supplementary material 1: Search keywords and output

Database	*Search strategy*	*Articles retrieved*
PubMed	*((((((((((eHealth[Title/Abstract]) OR Telemedicine[Title/Abstract]) OR Telehealth[Title/Abstract]) OR mHealth[Title/Abstract] OR* mobile health[Title/Abstract] *) OR web based[Title/Abstract]) OR internet[Title/Abstract] OR web-based[Title/Abstract]) OR Digital media[Title/Abstract]) OR short message service[Title/Abstract]) OR text message$[Title/Abstract]) OR videogame$[Title/Abstract]) OR Health game$[Title/Abstract] AND Type 2 Diabetes*	***624***
Psych Info	*(mHealth OR mobile Health OR eHealth OR Internet OR web based intervention OR mobile app OR web-based OR Digital media OR Telemedicine OR Telehealth OR Videogame OR Health game) AND (Type 2 Diabetes)*	***5***
Web of Science	*(eHealth OR mHealth OR mobile Health OR Telemedicine OR Telehealth OR web based intervention OR internet OR web-based OR Digital media OR short message service OR text message$ OR videogame$ OR Health game$) AND ("Type 2 Diabetes")*	***775***
